# Early-life environment and differences in costs of reproduction in a preindustrial human population

**DOI:** 10.1371/journal.pone.0207236

**Published:** 2018-12-12

**Authors:** Ilona Nenko, Adam D. Hayward, Mirre J. P. Simons, Virpi Lummaa

**Affiliations:** 1 Department of Environmental Health, Faculty of Health Sciences, Jagiellonian University Medical College, Krakow, Poland; 2 Moredun Research Institute, Pentlands Science Park, Bush Loan, Penicuik, Midlothian, United Kingdom; 3 Department of Animal and Plant Sciences, University of Sheffield, Sheffield, United Kingdom; 4 Bateson Centre, University of Sheffield, Sheffield, United Kingdom; 5 Department of Biology, University of Turku, Turku, Finland; Université de Sherbrooke, CANADA

## Abstract

Reproduction is predicted to trade-off with long-term maternal survival, but the survival costs often vary between individuals, cohorts and populations, limiting our understanding of this trade-off, which is central to life-history theory. One potential factor generating variation in reproductive costs is variation in developmental conditions, but the role of early-life environment in modifying the reproduction-survival trade-off has rarely been investigated. We quantified the effect of early-life environment on the trade-off between female reproduction and survival in pre-industrial humans by analysing individual-based life-history data for >80 birth cohorts collected from Finnish church records, and between-year variation in local crop yields, annual spring temperature, and infant mortality as proxies of early-life environment. We predicted that women born during poor environmental conditions would show higher costs of reproduction in terms of survival compared to women born in better conditions. We found profound variation between the studied cohorts in the correlation between reproduction and longevity and in the early-life environment these cohorts were exposed to, but no evidence that differences in early-life environment or access to wealth affected the trade-off between reproduction and survival. Our results therefore do not support the hypothesis that differences in developmental conditions underlie the observed heterogeneity in reproduction-survival trade-off between individuals.

## Introduction

Life-history theory predicts that investment in reproduction results in reduced future reproduction and survival because these traits share a pool of finite resources [1]. In support of such predictions, empirical studies have shown that mothers that skip reproduction or produce smaller broods in a given year have higher chances of survival to the next breeding season [2–6]. Yet, due to variation in access to resources between individuals or seasons [7], the best evidence for the reproduction-survival trade-off comes from experimental studies which have been able to manipulate either the reproductive effort or resource access during reproduction and link this to reduced maternal survival [8–10]. Nevertheless, even experimental studies often demonstrate large variation between populations, cohorts or individuals in the magnitude of the survival costs of reproduction [11, 12]. Such heterogeneity could be explained by variation between individuals in acquisition and allocation of available resources generating variable sized resource pools [13] and by interactions between genotype and environment [14] that can also lead to positive relationships between fitness traits in certain conditions.

Similarly, results from studies in humans measuring the survival costs of reproduction remain variable. While some studies have shown a negative relationship between number of children and maternal lifespan (e.g. [15–17]), some have shown positive associations [18, 19], or no association at all (e.g. [20, 21]). In addition, some studies on humans have only found the predicted negative association between reproduction and survival among the poorest mothers in the population, whereas wealthier women show no or positive associations [22, 23]. Interestingly, Wang and colleagues found that the relationship between the number of children born and lifespan differed among cohorts from the same population born in different years [24]. Historical data from Framingham, USA, showed generally positive correlations in cohorts born in years 1893 to 1907 and negative ones in those born from 1908 to 1913. The authors proposed that environmental perturbations experienced during reproductive years by the latter group such as the Great Depression and Second World War may explain the differences among cohorts, but the specific factors leading to differential cost of reproduction experienced by different cohorts were not studied on a cohort-specific basis.

One potential key factor responsible for differences between cohorts and individuals in their ability to cope with the cost of reproduction that has received little attention in humans [25] or other species [26] is variation in environmental conditions experienced during a mother’s own early development. Variation in early-life environment such as season of the mother’s birth [27, 28], population density [29], drought [30], spring temperature [31], or nutrition [32] at the time of the mother’s birth have been related to between-individual differences in survival and/or reproductive performance in many species [33, 34] including humans [35]. Suggested physiological mechanisms responsible for the relationship between early-life environment and later-life fitness include effects of poor early-life environment on rate of telomere loss [36], metabolic rate [37] and resistance to oxidative stress [38]. However, to our knowledge, there are only few studies in any species in which variation in early-life environment was analysed in relation to survival costs of reproduction. For example, it has been demonstrated in a wild Mauritius kestrel (*Falco punctatus*) population that females born in a lower-quality natal environment experience reduced age-specific survival, but the observed relationship was not driven by a short-term cost of reproduction [39]. In addition, experiments performed by Oksanen and colleagues determined that smaller birth size (possibly indicative of poor developmental conditions) and high breeding density in bank voles (*M*. *glareolus*) increased survival costs of reproduction in later life [26].

Effects of early-life environments on the ability to cope with the cost of reproduction are plausible, because environmental effects experienced during development can extend into and influence the whole length of adult life, a phenomenon which is already well-established, especially in humans [40], through changes in body structure and physiology. Note also that these effects do not only include severe deprivation (e.g. famine in humans), but early environment effects on later health can also be detected within the ‘normal’ range of weight at birth [41]. In humans, a more favourable early-life environment has been shown to have a positive association with lifetime reproductive success [42, 43] and longevity [44–46]. Meanwhile, a poorer early-life environment has been associated with: increased risk of dying from infectious diseases in early adulthood [47] and from complications of giving birth [48]; increased risk of developing cardiovascular diseases, obesity, and non-insulin dependent diabetes mellitus [40, 49, 50]; and reduced late-life reproduction and survival [51]. However, although such previous studies have highlighted how early-life environment impacts on both reproduction and longevity in humans, no studies have investigated its impact on the actual trade-off between reproduction and survival.

In this study, we test the hypothesis that individuals who experienced a poor early-life environment should show larger survival costs of reproduction than those experiencing a better early-life environment. To this end, we used individual-based life-history data collected from church records for 1 899 Finnish women born 1751–1850, combined with historical records of annual crop yields, spring temperature and infant mortality in early life as detailed proxies for their early-life environment. First, we quantify variation in the relationship between reproduction and survival among the 81 birth cohorts included in this study. Second, we determine whether variation in the cost of reproduction among cohorts can be explained by the early developmental conditions experienced by individuals. We test the effect of both the number of born and raised offspring on maternal survival in order to include the costs incurred from pregnancy (offspring born) and parental care to those offspring (offspring raised to adulthood). We further test whether the socio-economic status of the studied women modified such effects, predicting that women from the lowest socio-economic group should be most adversely affected by poor developmental conditions and should thus exhibit the largest survival cost of reproduction.

## Materials and methods

### Study population and data collection

Study was approved by the Jagiellonian University Research Ethics Committee. We used demographic data collected by the Lutheran church in Finland from the 18th century onwards. Our database was compiled using church registers of births, movements, marriages and deaths in five Finnish parishes: Hiittinen, Kustavi, Tyrvää, Rymättylä and Ikaalinen. The population was strictly monogamous; women may have remarried only if their husband died, and both divorce and adultery were outlawed [52] and extra-marital paternity was very low [53]. The main source of livelihood was farming, with around 60% of the energy consumed by working people contributed by rye alone, 20% from potatoes and barley [54], and the remainder from meat, fish and dairy products [55]. In general, the standard of living was low in the studied population and climatic conditions in Finland were challenging, resulting in substantial between-year variation in food availability [56]. We used data collected on women born between 1751 and 1850 (the years used differ for some early-life environment measures). Women included in the study were therefore at least 50 years old in 1900 and experienced natural fertility during their reproductive lifespan, since the demographic transition to lower child mortality rates and female fertility began from the 1880s onwards but largely occurred only in the 1900s [57, 58]. These women gave birth on average to 4.92 children (range = 0–14; SD = 3.12); 50% of women gave birth to 5 children or more. 65% of the children of these women survived until 15 years of age and 50% of the women in the sample lived for at least 63 years. This selection resulted in a total of 1 899 women for whom we knew their socio-economic status and their lifetime number of children born. The individuals were classified into two socio-economic classes, which differed greatly in their access to resources, based on their husband’s occupation: a rich class included farm owners and merchants, craftsmen and tenant farmers, and a poor class consisted of crofters and labourers [59]. Our analyses control for potential confounding factors such as shared family effects and variation due to birth order, geographic location and year. Three proxies of the quality of the early-life environment were used: rye yield, annual spring temperature, and yearly infant mortality in the population around the time of each individual’s birth as justified below.

#### Rye yield

Firstly, rye yield was used as a proxy of quality of the early-life environment. Annual harvest success was quantified as the amount of grain harvested as a multiple of the quantity sown (‘yield’ from herein), a measure which is unbiased by variation in planting effort and population size. In addition, low grain yields have been found to be associated with documented famines [60]. Although grain yields do not directly reflect individual food availability, this measure is relative to the success compared to other years and reflects year-to-year variation in harvest quality, which varied considerably during the study period [61]. Birth year grain figures in the study population predict later-life survival [46, 51] and reproductive success [42]. Further, current grain figures influence reproductive rate [62] and instantaneous survival [61].

#### Spring temperature

Second, we used spring temperature as a measure of conditions around the time of birth, which is associated with the severity of the winter: higher spring temperature is associated with a less severe winter and a more successful rye harvest [63]. It was reconstructed using standard multiproxy techniques from historical data including sea ice break-up and plant phenology which explained a large proportion of the variance in observed February–June temperatures in south-west Finland [64]. We have previously shown in the pre-industrial Finnish population studied here that spring temperature is negatively related to child and adulthood mortality [65].

#### Infant mortality

Finally, mean infant mortality was calculated as the proportion of infants born in each parish each year who died in their first year of life. This constitutes a demographic measure of environmental quality and has been used in the context of environmental quality in ecological studies of animals [66, 67], another study on humans [68], and a previous study on the Finnish population studied here [65], which showed that individuals born in years with high infant mortality experienced higher mortality later in life. In this study, data were taken from census records but gaps in such record-keeping meant that the data were not available for all years in each parish that the demographic records cover.

### Statistical analysis

All analyses were conducted in R ver. 3.4.2 [69]. First, for illustrative purposes, we calculated a Spearman correlation, using the function *cor*.*test*, between the number of children born and longevity for individuals in each birth cohort separately (birth cohorts with <10 individuals excluded, in total 1695 individuals in 81 cohorts were analysed) to visualize variation in the cost of reproduction to survival over our entire study period (indicated by a negative correlation between reproduction and lifespan). We included women with known date of death and who completed their reproductive lifespan (survived at least to 50 years of age). Women who died before 50 years of age were excluded only for this correlation analysis; all women, regardless of their lifespan, were included in the further analyses (described below) to avoid selection bias resulting from mortality during reproductive years [70].

Second, to test whether environmental conditions experienced in early life modify the cost of reproduction to individual survival (i.e. the hazard between the number of children born and mortality at a given time step) we used mixed-effects Cox right-censored regression models with number of children born as an accumulating time-dependent covariate and survival as response variable using the package *‘coxme’* [71]. We tested whether the effect of the number of children on survival at each time interval was modified by the early-life environment of the individual by fitting an interaction between the measure of early-life environment (see below) and the number of children born. This method combines a few important strengths. First of all, it allowed us to take into account time-independent (e.g. socio-economic status) and time-dependent variables (e.g. number of children born up to each age). Second, the method allows for inclusion of censored individuals, that is, individuals that have not been followed until the end of the study period (for example, because of migration). Third, it allowed us to include random effects in order to account for the dependency owing to shared family or the same year of the study.

We further tested whether early developmental conditions modified the cost of reproduction on survival when the cost of parental care (in addition to production of offspring) was included in the total cost of reproduction. To this end, the effect of lifetime number of children who survived to fifteen years of age on maternal survival was analysed with mixed-effects Cox right-censored regression models in order to also test for costs of parental care involved in raising children, which has been suggested to be higher than costs of bearing children [72].

### Early-life environment and children–survival relationship

#### (i) Rye yield

Rye yields were available for Ikaalinen and Tyrvää for 1804–1850. The corresponding survival and reproduction data contained 5007 intervals from 759 women (501 with known date of death and 258 censored). The data were encoded using (start, stop] form of the model [73]. The first interval in the dataset for a woman started with 0 (date of birth) and finished with age at first reproduction which corresponds with 1 in the variable ‘number of children born’. Each interval stopped when another child was born and the last record stopped with age at death. For those individuals with unknown age at death, ‘date of last appearance’ (as recorded by the church e.g. in migration registers) was used in the models and those individuals were considered as right-censored. We tested whether rye yield experienced in early life modified the relationship between number of children born up to the age at which survival was being assessed and subsequent survival. Rye was harvested in autumn [74] and therefore women were assumed to eat rye from the previous year during January-August and from the current year since September. We calculated mean rye yield from a period which covers pregnancy and first year of life (Figure A in S1 File). Thus, for women born in January to May, the three-year mean was calculated from the values in the two years previous to the year of birth and the year of birth; for women born between June and August, the two-year mean was calculated from the year before and year of birth; for women born between September and December, the three-year mean was calculated from the year before birth, the year of birth, and the year after birth.

We took a hypothesis-testing approach by building four hypothesis-specific sets of models to reduce a high type I error rate resulting from model selection procedures [75]. Firstly, we tested the effect of the number of children born up to the age at which survival was being assessed on mortality risk as linear (Hypothesis 1) and quadratic (Hypothesis 2) terms. Then, we fitted three two-way interactions: *(i)* early-life rye yield * number of children born to test the prediction that the negative effect of bearing children will be more pronounced among those experiencing poor early-life environment *(ii)* number of children born * socio-economic class to test the effect of having children on survival among women with different socio-economic class, and *(iii)* early-life rye yield * socio-economic class in order to test the prediction that negative consequences of early-life environment would be most pronounced among women from the poorest, landless class (Hypothesis 3). Finally, we fitted a three-way interaction between early-life rye yield, number of children born and socio-economic class to test the prediction that the negative effect of poor early-life environment on the cost of reproduction was largest among the poorest socio-economic class (Hypothesis 4). Whenever the quadratic effect of children born was significant this term was further used in interactions. In all models, we included: mean rye yield around birth (as a linear covariate), twin status (as a categorical variable with two levels [76]); birth order (as a categorical variable with two levels: firstborns vs. laterborns [77]); birth parish (as a categorical variable with five levels); social status (as a categorical variable with two levels) [59]. Maternal identity and birth year were fitted as random effects to account for the dependency owing to shared family (closely related genetically and phenotypically) or the same year.

In order to test the costs of parental care on survival, the lifetime number of children surviving to 15 years of age was analysed in separate models using mixed-effects Cox right-censored regression models. Due to different intervals in between children who survived until 15 years of age, this variable was not tested as time-varying covariate. Instead, we used the total number of children surviving to 15 years of age. The procedure of building the models was the same as in models with number of children born. The aassumption of proportional hazards was not formally checked because there is no test of proportionality currently available for the mixed-effects Cox models [71].

#### Spring temperature

Information about spring temperature was available for all five parishes: Hiittinen, Kustavi, Tyrvää, Rymättylä, Ikaalinen for the period 1751–1850. The corresponding survival and reproduction data contained 1 899 women (1600 with known date of death and 299 right-censored) excluding individuals who had unknown parental identity or socio-economic status; overall, we analysed 11 072 intervals from 1 899 women. Spring temperature is highly correlated with harvest quality [63], so mean spring temperature during pregnancy and the first year of life was calculated, taking into account that harvest time was in September. We fitted the same models as for rye yield, but replacing rye yield with spring temperature in all cases.

#### Infant mortality

Finally, the models were repeated with population infant mortality as a measure of early-life environment. Information about infant mortality was available for five parishes: Hiittinen, Kustavi, Tyrvää, Rymättylä and Ikaalinen, for the period 1751–1850, with only some years missing due to damage of the records [65]. Two out of five parishes have complete records for each year of the entire study period (Tyrvää and Ikaalinen). In Rymättylä there is missing information in one year, in Kustavi information on infant mortality was missing for 10 years, and in Hiittinen there is no information for 22 years. The corresponding survival and reproduction data comprised 10 233 intervals from 1753 women (1467 with known date of death and 286 censored). The three-year mean centred value of infant mortality around the individual’s year of birth was calculated to capture environmental quality around pregnancy and the first year of life for every woman. Again, we fitted the same models as for rye yield, but replacing rye yield with infant mortality in all cases.

## Results

**Fig 1:**
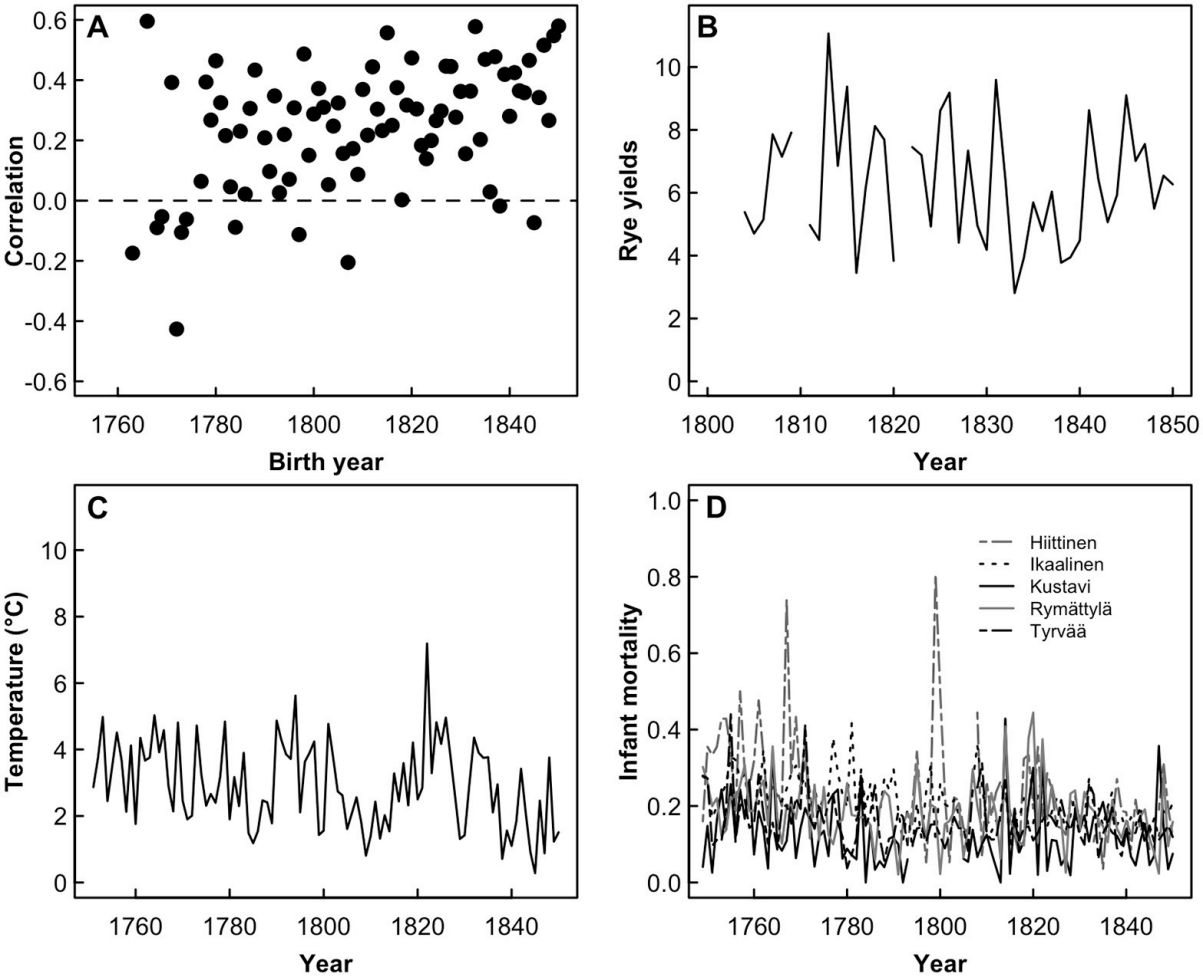
Variation in the relationship between children born and lifespan, and environmental conditions in the study period (1751–1850); (a) the relationship between number of children born and post-reproductive lifespan among 81 birth cohorts (1751–1850) varied between r_s_ = -0.43 and r_s_ = 0.6, but is on average positive, indicating higher reproduction is associated with longer lifespan; (b) rye yields varied between years across the study period; (c) spring temperature estimated using multiproxy reconstruction varied substantially between years during the studied period; (d) the proportion of children who were born in a given year and died before the age of 1 (infant mortality) varied between years in the five studied parishes between 0.0 and 0.81.

As illustrated in Fig 1a, considerable variation existed between birth cohorts in the correlation between reproduction and longevity among post reproductive women: while 14% of all cohorts show negative relationships ranging from weak (r_s_ = -0.02) in 1838 to strong (r_s_ = -0.43) in 1772, some cohorts showed strongly positive correlations between reproduction and longevity (e.g. r_s_ > 0.56 in 1766, 1833, 1850). Consequently, cohorts vary considerably in how reproduction is associated with longevity with a positive trend over time (β = 0.004, s.e. = 0.001, p < 0.001). We next sought to determine whether this variation was driven by early-life environment.

There was also considerable between-year variation in annual crop yields, spring temperature and population infant mortality during the study period [Coefficient of variation (CV): 0.30, 0.44, and 0.53 respectively (Fig 1b–1d)]. For example, annual rye yield varied 5-fold between years (Fig 1b); spring temperature varied by over 6°C between the coldest (1845) and warmest (1822) years (Fig 1c); and whilst 97% of all infants born in 1835 survived to age 1 in Hiittinen, there were years during the study period when >50% of the infants born died before their first birthday (Fig 1d). Spring temperature declined (linear model [lm]: β = -0.012 ± 0.004, p = 0.005) and infant mortality increased (generalised linear model with quasibinomial distribution: β = 0.004 ± 0.001, p < 0.001) somewhat across the study period, but annual rye yield did not consistently increase or decrease (lm: β = -0.011 ± 0.021, p = 0.589).

**Fig 2:**
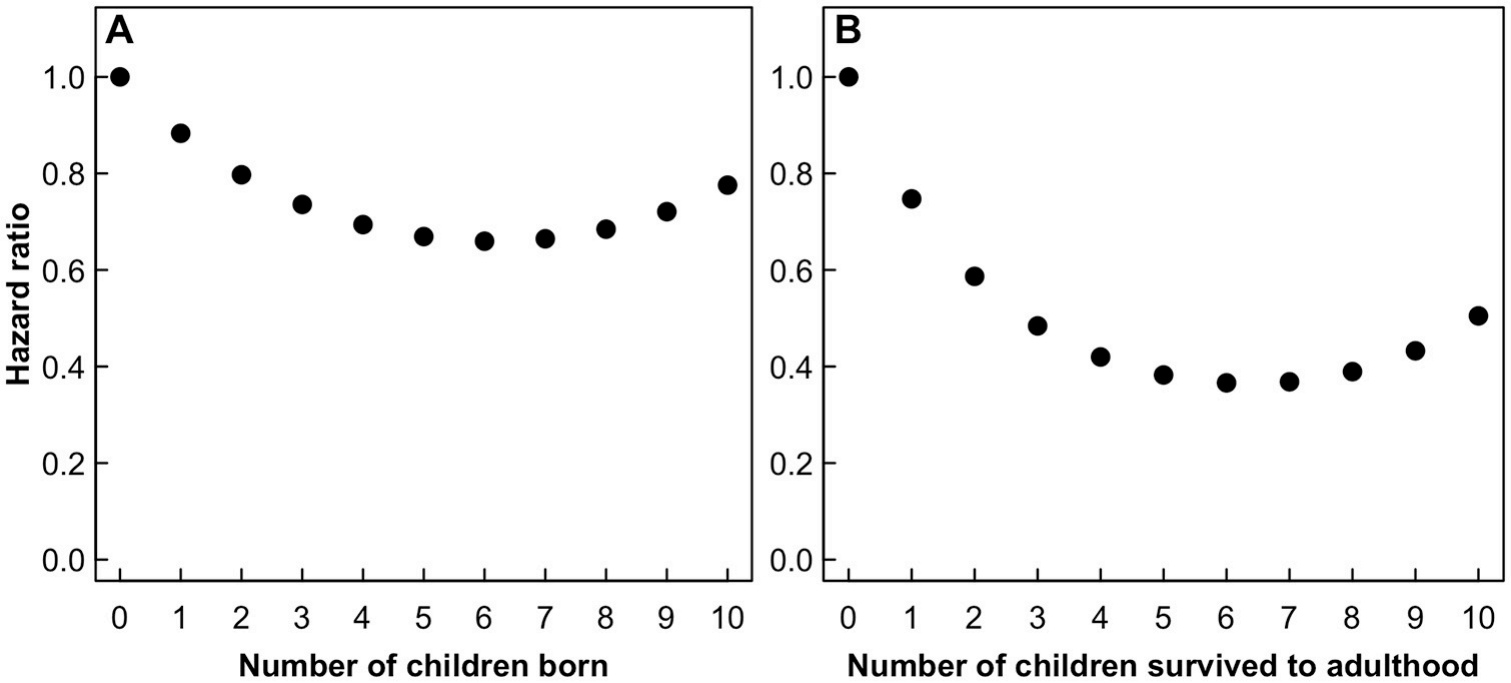
Quadratic relationship between number of children and maternal mortality risk. The predicted values of hazard from the model across the range of the data. (a) quadratic relationship between number of children born and maternal mortality risk (Hypothesis 2); (b) quadratic relationship between number of children survived to 15 years of age and maternal mortality risk (Hypothesis 2).

Firstly, we examined if the number of children was associated with individual survival probability at each step across lifespan. Overall, there was no linear association between number of children and probability of survival following each reproduction (Hypothesis 1: hazard 0.99; 95% confidence interval 0.95–1.02; p = 0.40), but there was significant quadratic association. On average, women had lower mortality risk up to seven children and then mortality risk started to increase (Hypothesis 2: linear hazard 0.87; 95% confidence interval 0.79–0.97; p = 0.008, quadratic hazard 1.01; 95% confidence interval 1.00–1.02; p = 0.01; Fig 2a). We therefore investigated whether the between-individual variation in the cost of reproduction is associated with different early-life environment experienced by those individuals. We did not observe differences between the rich and the poor women in terms of lower probability of survival following each reproductive event (poor * children born, β = 0.008 ± 0.146, z = 0.05, p = 0.96, Table A in S1 File, model 1). Further, we found no support for the prediction that those experiencing poor early-life environment, as measured by rye yield around the year of birth, would pay larger costs of reproduction in terms of lower probability of survival following each reproduction (children born * rye, β = 0.0075 ± 0.048, z = 1.54, p = 0.12, Table A in S1 File, model 1). We also tested whether any such effects of early conditions would be most pronounced among women from the poor class, but we did not observe a difference in survival between rich and poor women depending on their early-life environment (poor * rye, β = 0.103 ± 0.111, z = 0.93, p = 0.35, Table A in S1 File, model 1). Moreover, the effect of rye yield on the association between number of children and age-specific survival did not differ between the poor and the rich women (children born * poor * rye, β = -0.160 ± 0.166, z = -0.97, p = 0.33, Table A in S1 File, model 1a).

Next, we tested whether the number of children who survived to adulthood (cost of parental care) was associated with mother’s probability of survival. There was a linear association between number of children and maternal survival (Hypothesis 1: hazard 0.89; 95% confidence interval 0.85–0.93; p < 0.001). Every additional child that survived to adulthood was associated with an 11% lower probability of mother’s mortality. There was also a quadratic association between the number of children surviving and maternal survival, indicating that the number of children that survived to adulthood was associated with lower mortality, but the effect weakened as the number of children increased (Hypothesis 2: linear hazard 0.73; 95% confidence interval 0.65–0.82; p < 0.001, quadratic hazard 1.03; 95% confidence interval 1.01–1.04; p < 0.001; Fig 2b). We then examined whether variation in the cost of parental care was related to different early conditions experienced by different mothers (Table B in S1 File). Firstly, we did not find evidence for a higher cost of parental care for women with low-quality early-life environments (no. of children 15yrs * rye, β = 0.057 ± 0.054, z = 1.06, p = 0.29, Table B in S1 File, model 1). The effect of early-life rye yield remained similar for women from both social groups (poor * rye, β = 0.119 ± 0.119, z = 1.00, p = 0.32, Table B in S1 File, model 1), but women from the poor class had lower survival probability with increasing number of children surviving to 15 years of age comparing to women from the rich class (poor * no. of children 15yrs, β = -0.313 ± 0.151, z = -2.07, p = 0.04, Table B in S1 File, model 1). Finally, there was no support for the hypothesis that poor early-life environment was associated with the highest cost of reproduction to maternal survival among those woman from the poorest socioeconomic background (no. of children 15yrs * poor * rye, β = -0.173 ± 0.174, z = -0.99, p = 0.32, Table B in S1 File, model 1a).

The results remained qualitatively unchanged when either spring temperature or infant mortality rate was used as an indicator of early-life environment instead of rye yields (electronic supplementary materials, Table C-F in S1 File).

## Discussion

In this study, we tested whether women experiencing poor early-life environmental conditions displayed a stronger trade-off between investment in reproduction and survival compared to individuals experiencing a more favourable early-life environment. We used detailed demographic data collected from a preindustrial Finnish population experiencing natural fertility and mortality, combined with three measures of early-life environmental quality: spring temperature, infant mortality and rye yields. Early-life environmental conditions did not modify the relationship of maternal survival with either the number of children produced by a female or the number of children raised to adulthood. Additionally, we did not find evidence that women from the lowest socio-economic class were most adversely affected by poor early-life environments. Below, we discuss these results in relation to demographic, anthropological and reproductive ecology literature.

We found a U-shaped relationship between both children born and survived to adulthood and maternal mortality risk. Women’s mortality risk was reduced by each additional birth up to seven children, after which point their mortality risk started to increase again, but few women in our population had this many children in their lifetime. Similar results were described in groups of women from Norway and Israel where women having 2–4 children had lower mortality compared to women with 0–1 children, but mortality risk started to increase again for women with 5 children and more [16, 78]. Taken together, these findings suggest that although reproduction is known to be costly, costs detrimental for maternal health and consequently shortening their lifespan are commonly seen only among women with many children. Thus, detecting this trade-off may be hard because few women in the population have such numbers of children that are detrimental for their health. Moreover, possible improvements in the society’s sanitation, health and access to resources over the studied time period could have reduced the biological necessity for women to trade-off reproduction and survival.

We found no support for our hypothesis that a poor early-life environment would lead to a stronger trade-off between reproduction and survival. Although a poor early-life environment has been related to reduced survival and/or reproductive performance in many species [33, 34] including humans [35], only few previous studies in any species has investigated whether poor early-life environment increases the survival costs of reproduction in females [26, 39]. We hypothesized that such effects of early-life environments on the ability to cope with the cost of reproduction would be likely, as an increasing number of studies have shown that early conditions can permanently alter body structure and physiology in a way that decreases both reproductive success and longevity (i.e. fitness) in adulthood [43, 44] (but see [79]), including this study population [42, 46, 51]. In contrast to these predictions, however, we found no evidence to support the hypothesis that a poor early-life environment increased the survival costs of reproduction in pre-industrial women. Similar results were found in wild Mauritian kestrels [39]. This contradicts findings from the bank voles, in which poor early-life environment measured by smaller size and high breeding density at birth increased survival costs of reproduction in adulthood [26]. A possible explanation for this discrepancy could be that long-lived species evolved a slow life-history strategy and favour their own survival over producing (too many) offspring in order to obtain higher fitness [80]. This is in contrast to short-lived species, which have evolved a fast life-history strategy where individuals reproduce whenever there is a chance to do so. Bank voles are short-lived and on average give birth up to four litters during the season [81], while humans are long-lived and more carefully adjust their reproductive output to their condition and may only reproduce when “they can afford to do so”, avoiding strong detriments to survival. Ovarian function is very sensitive to energy supply and this sensitivity is thought to protect maternal condition and to optimize a woman’s lifetime reproductive output [82]. Therefore, reproductive suppression through ovarian function can lengthen the inter-birth intervals and allows women to improve their own nutritional status before their next pregnancy. Thus, it is possible that we did not detect effects of developmental conditions on the relationship between reproductive output and survival, because pregnancy only happened at a time when energy conditions were good enough to maintain both good maternal condition and pregnancy. This strategy may also enhance child fitness since good maternal condition is associated with higher birth weight and increased child survival probability [83]. Hence, the apparent lack of a negative relationship between the total number of children born and maternal lifespan observed in many populations [84] could be due to the fact that the number of pregnancies is adjusted to environmental conditions without detrimental effects on mothers. Nevertheless, this explanation is not completely satisfactory as women in some populations [16, 85] or in some cohorts (this study, [24]) exhibit reproductive rates that bear costs on their survival, and our findings therefore call for further investigation in other populations and ecologies.

Several limitations must be considered when assessing our results. Firstly, our results could have been different if we would have been able to assess an individual’s level of resources (not available for historical populations) rather than using cohort-level differences as a proxy. Here we used three different measures of early-life environment, all of which have been used in previous studies on this population [42, 51, 65]. Rye yields around birth were shown to have a positive association with reproductive success, especially in women from the poorest socio-economic class [42]. Landless women exposed to higher rye yields around birth had higher probability of reproducing in their lifetime and had a higher proportion of children surviving to adulthood compared to landless women born in low rye yields [42]. Lower spring temperature around birth has been shown to be associated with higher mortality in childhood and adulthood in our study population [65]. Finally, high infant mortality around birth is associated with increased mortality in childhood in our population, but the effect apparently does not persist into adulthood [65]. Overall, taking into account the above findings, we would argue that spring temperature, infant mortality, and grain-yield data do reflect meaningful variation in environmental conditions around birth which have significant effects on fitness in our population, although they apparently do not mediate between-individual differences in the costs of reproduction on maternal survival. Possibly, conditions which women experience during reproductive years are more important for creating variation in costs of reproduction than environment experienced early in life. However, such scenarios fit poorly with the observed differences between birth cohorts, with each cohort living and reproducing across a range of later-life years and conditions, and yet displaying distinct differences according to their birth cohort. Second, previous studies on humans have shown that the costs of reproduction can be offspring sex-specific, with sons costing more to maternal longevity than daughters ([20], but see [86]). We did not focus on such sex-specific costs here, but instead measured the costs of reproduction by the number of born or raised offspring regardless of their sex. Although adjusting for the sex of each birth would allow to address some of the between-birth variation in the costs of reproduction to mothers, it is unlikely that the between-cohort differences in the cost of reproduction we observe (Fig 1a) would be driven by differential lifetime sex allocation between women born in different years.

In conclusion, despite using a highly detailed database on individual reproductive histories on several hundred women experiencing natural fertility, we did not find evidence that environmental conditions experienced during early life modified the relationship between reproduction and survival. Many previous studies measuring the cost of reproduction on survival have used lifetime reproductive success as a measure of reproductive investment, and contrasted this with post-reproductive survival (e.g. [15, 23, 86]). Potential problems with this approach are that *(i)* it means excluding women who could suffer detrimental cost of reproduction during reproductive years and as a consequence die young (before 50 years of age), and *(ii)* this approach does not consider the contrasting effects of bearing children versus raising them to independence. Here, we addressed these issues directly by fitting the number of children born as a time-varying covariate and analysing the effects of both the number of births and number of children survived to adulthood on maternal survival. Despite apparent variation between cohorts in the trade-off between reproduction and survival we were unable to determine the drivers of this variation. Therefore, we hope that such variation as well as the predictions of the hypothesis studied here continue to be tested using a broad range of approaches and making use of available data, since only then we will increase our understanding of the causes of between-individual differences in reproduction-survival trade-offs, and any role of the early-life environment therein.

## Supporting information

S1 File File containing Figure A explaining conception behind calculation of mean rye for a period which covers pregnancy and first year of life and Tables A-F with mixed-effects Cox regression models of the effects of number of children born and survived to adulthood (respectively) and early-life environment (rye yields, spring temperature, infant mortality) on maternal survival. (DOCX)

S1 Dataset Spearman correlation results. (CSV)

S2 Dataset Rye yields data. (CSV)

S3 Dataset Spring temperature data. (CSV)

S4 Dataset Infant mortality data. (CSV)

S5 Dataset Individual-level information for cox models on number of children born and maternal survival. (CSV)

S6 Dataset Individual-level information for cox models on number of children survived to adulthood and maternal survival. (CSV)
