# Supplementary material for: Early-life environment and differences in costs of reproduction in a preindustrial human population
Source: PLoS One. 2018 Dec 12;13(12):e0207236. doi: 10.1371/journal.pone.0207236 (PMC6291071; doi:10.1371/journal.pone.0207236)
Supplement: S1 File — (DOCX) [file pone.0207236.s001.docx]

**Early-life environment and differences in costs of reproduction in a preindustrial human population**

**Ilona Nenko^1*^, Adam D. Hayward^2^, Mirre J. P. Simons^3,4^, Virpi Lummaa^5^**

Figure A. Graph explaining conception behind calculation of mean rye for a period which covers pregnancy and first year of life. September was the harvest month. Thus, for example for women born in January to May in 1801, the three-year mean was calculated from the values in the two years previous to the year of birth and the year of birth (1799-1801); for women born between June and August, the two-year mean was calculated from the year before and year of birth (1800-1801); for women born between September and December, the three-year mean was calculated from the year before birth, the year of birth, and the year after birth (1800-1802).

**Rye yield**

**Table A.** A mixed-effects Cox regression model of the effects of number of children born and early-life environment – rye yield - on maternal survival. Model 1 shows results for predictions of Hypothesis 3 and Model 1a shows results for predictions of Hypothesis 4. β = Cox regression coefficient; s.e. (β) standard error of Cox regression coefficient

| **Variable** | **β** | **s.e. (β)** | | **z-value** | **p-value** | **β** | **s.e. (β)** | **z-value** | **p-value** |  |
| --- | --- | --- | --- | --- | --- | --- | --- | --- | --- | --- |
|  | *Model 1* | | | | | *Model 1a* | | | | |
| *Fixed effects* |  |  | |  |  |  |  |  |  |  |
| Birth status (singleton) | 0.000 |  | |  |  | 0.000 |  |  |  |  |
| Birth status (twin) | -0.284 | 0.322 | | -0.88 | 0.38 | -0.284 | 0.323 | -0.88 | 0.38 |  |
| Birth order (first) | 0.000 |  | |  |  | 0.000 |  |  |  |  |
| Birth order (subsequent) | -0.167 | 0.107 | | -1.56 | 0.12 | -0.169 | 0.107 | -1.57 | 0.12 |  |
| Parish, Ikaalinen | 0.000 |  | |  |  | 0.000 |  |  |  |  |
| Parish, Tyrvää | 0.120 | 0.115 | | 1.04 | 0.30 | 0.112 | 0.116 | 0.96 | 0.34 |  |
| Social class (rich) | 0.000 |  | |  |  | 0.000 |  |  |  |  |
| Social class (poor) | -0.552 | 0.769 | | -0.72 | 0.47 | -2.394 | 2.083 | -1.15 | 0.25 |  |
| Children born | -0.612 | 0.311 | | -1.96 | 0.05 | -0.724 | 0.334 | -2.17 | 0.03 |  |
| Children born^2 | 0.045 | 0.027 | | 1.63 | 0.10 | 0.053 | 0.029 | 1.85 | 0.06 |  |
| Rye | -0.254 | 0.126 | | -2.01 | 0.04 | -0.297 | 0.137 | -2.16 | 0.03 |  |
| Social class (rich) : children born | 0.000 |  | |  |  | 0.000 |  |  |  |  |
| Social class (poor) : children born | 0.008 | 0.146 | | 0.05 | 0.96 | 1.005 | 1.044 | 0.96 | 0.34 |  |
| Social class (rich) : children born^2 | 0.000 |  | |  |  | 0.000 |  |  |  |  |
| Social class (poor) : children born^2 | 0.001 | 0.014 | | 0.04 | 0.97 | -0.091 | 0.103 | -0.89 | 0.37 |  |
| Children born : rye | 0.075 | 0.048 | | 1.54 | 0.12- | 0.092 | 0.052 | 1.77 | 0.08 |  |
| Children born^2 : rye | -0.005 | 0.004 | | -1.24 | 0.21 | -0.007 | 0.004 | -1.49 | 0.14 |  |
| Social class (rich) : rye | 0.000 |  | |  |  | 0.000 |  |  |  |  |
| Social class (poor) : rye | 0.103 | 0.111 | | 0.93 | 0.35 | 0.397 | 0.326 | 1.22 | 0.22 |  |
| Social class (rich) : children born : rye | - | - | | - | - | 0.000 |  |  |  |  |
| Social class (poor) : children born : rye | - | - | | - | - | -0.160 | 0.166 | -0.97 | 0.33 |  |
| Social class (rich) : children born^2 : rye | - | - | | - | - | 0.000 |  |  |  |  |
| Social class (poor) : children born^2 : rye | - | - | | - | - | 0.015 | 0.016 | 0.90 | 0.37 |  |
| *Random effects* | Variance (SD) | |  |  |  | Variance (SD) |  |  |  |  |
| Maternal ID | 0.06 (0.25) |  | |  |  | 0.07 (0.26) |  |  |  |  |
| Birth year | 0.02 (0.14) |  | |  |  | 0.02 (0.14) |  |  |  |  |

**Table B.** A mixed-effects Cox regression model of the effects of number of children survived to adulthood and early-life environment – rye yield - on maternal survival. Model 1 shows results for predictions of Hypothesis 3 and Model 1a shows results for predictions of Hypothesis 4. β = Cox regression coefficient; s.e. (β) standard error of Cox regression coefficient

| **Variable** | **β** | **s.e. (β)** | | **z-value** | **p-value** | **β** | **s.e. (β)** | **z-value** | **p-value** |  |
| --- | --- | --- | --- | --- | --- | --- | --- | --- | --- | --- |
|  | *Model 1* | | | | | *Model 1a* | | | | |
| *Fixed effects* |  |  | |  |  |  |  |  |  |  |
| Birth status (singleton) | 0.000 |  | |  |  | 0.000 |  |  |  |  |
| Birth status (twin) | -0.311 | 0.332 | | -0.94 | 0.35 | -0.312 | 0.332 | -0.94 | 0.35 |  |
| Birth order (first) | 0.000 |  | |  |  | 0.000 |  |  |  |  |
| Birth order (subsequent) | -0.177 | 0.111 | | -1.60 | 0.11 | -0.180 | 0.111 | -1.62 | 0.10 |  |
| Parish, Ikaalinen | 0.000 |  | |  |  | 0.000 |  |  |  |  |
| Parish, Tyrvää | 0.108 | 0.122 | | 0.89 | 0.38 | 0.116 | 0.122 | 0.95 | 0.34 |  |
| Social class (rich) | 0.000 |  | |  |  | 0.000 |  |  |  |  |
| Social class (poor) | -0.419 | 0.772 | | -0.54 | 0.59 | -1.181 | 1.551 | -0.76 | 0.45 |  |
| Children 15yrs | -0.621 | 0.348 | | -1.79 | 0.07 | -0.691 | 0.375 | -1.84 | 0.07 |  |
| Children 15yrs^2 | 0.045 | 0.041 | | 1.11 | 0.27 | 0.056 | 0.043 | 1.30 | 0.19 |  |
| Rye | -0.169 | 0.105 | | -1.61 | 0.11 | -0.178 | 0.114 | -1.57 | 0.12 |  |
| Social class (rich) : children 15yrs | 0.000 |  | |  |  | 0.000 |  |  |  |  |
| Social class (poor) : children 15yrs | -0.313 | 0.151 | | -2.07 | 0.04 | 0.720 | 1.079 | 0.67 | 0.50 |  |
| Social class (rich) : children 15yrs^2 | 0.000 |  | |  |  | -0.125 | 0.142 | -0.88 | 0.38 |  |
| Social class (poor) : children 15yrs^2 | 0.040 | 0.020 | | 2.04 | 0.04 |  |  |  |  |  |
| Children 15yrs : rye | 0.057 | 0.054 | | 1.06 | 0.29 | 0.068 | 0.058 | 1.17 | 0.24 |  |
| Children 15yrs^2 : rye | -0.004 | 0.006 | | -0.67 | 0.50 | -0.006 | 0.007 | -0.88 | 0.38 |  |
| Social class (rich) : rye | 0.000 |  | |  |  | 0.000 |  |  |  |  |
| Social class (poor) : rye | 0.119 | 0.119 | | 1.00 | 0.32 | 0.246 | 0.247 | 1.00 | 0.32 |  |
| Social class (rich) : children 15yrs : rye | - | - | | - | - | 0.000 |  |  |  |  |
| Social class (poor) : children 15yrs : rye | - | - | | - | - | -0.173 | 0.174 | -0.99 | 0.32 |  |
| Social class (rich) : children 15yrs^2: rye | - | - | | - | - | 0.000 |  |  |  |  |
| Social class (poor) : children 15yrs^2: rye | - | - | | - | - | 0.028 | 0.023 | 1.20 | 0.23 |  |
| *Random effects* | Variance (SD) | |  |  |  | Variance (SD) |  |  |  |  |
| Maternal ID | 0.14 (0.37) |  | |  |  | 0.14 (0.37) |  |  |  |  |
| Birth year | 0.01 (0.09) |  | |  |  | 0.01 (0.10) |  |  |  |  |

**Spring temperature**

There was neither linear (Hypothesis 1: hazard 1.00, 95% confidence interval 0.98 – 1.02, P = 0.76) nor quadratic (Hypothesis 2: hazard 1.00, 95% confidence interval 0.99 – 1.01, P = 0.66) association between number of children born and survival probability at each time step.

**Table C.** A mixed-effects Cox regression model of the effects of number of children born and early-life environment – spring temperature - on maternal survival. Model 1 shows results for predictions of Hypothesis 3 and Model 1a shows results for predictions of Hypothesis 4. β = Cox regression coefficient; s.e. (β) standard error of Cox regression coefficient

| **Variable** | **β** | **s.e. (β)** | | **z-value** | **p-value** | **β** | **s.e. (β)** | **z-value** | **p-value** |  |
| --- | --- | --- | --- | --- | --- | --- | --- | --- | --- | --- |
|  | *Model 1* | | | | | *Model 1a* | | | | |
| *Fixed effects* |  |  | |  |  |  |  |  |  |  |
| Birth status (singleton) | 0.000 |  | |  |  | 0.000 |  |  |  |  |
| Birth status (twin) | -0.105 | 0.131 | | -0.80 | 0.42 | -0.103 | 0.131 | -0.78 | 0.43 |  |
| Birth order (first) | 0.000 |  | |  |  | 0.000 |  |  |  |  |
| Birth order (subsequent) | -0.110 | 0.064 | | -1.74 | 0.08 | -0.110 | 0.064 | -1.72 | 0.09 |  |
| Parish, Hiittinen | 0.000 |  | |  |  | 0.000 |  |  |  |  |
| Parish, Ikaalinen | 0.096 | 0.085 | | 1.13 | 0.26 | 0.096 | 0.085 | 1.14 | 0.26 |  |
| Parish, Kustavi | 0.271 | 0.099 | | 2.73 | 0.006 | 0.273 | 0.099 | 2.74 | 0.006 |  |
| Parish, Rymättylä | 0.248 | 0.114 | | 2.18 | 0.03 | 0.246 | 0.114 | 2.16 | 0.03 |  |
| Parish, Tyrvää | 0.223 | 0.098 | | 2.27 | 0.02 | 0.223 | 0.098 | 2.27 | 0.02 |  |
| Social class (rich) | 0.000 |  | |  |  | 0.000 |  |  |  |  |
| Social class (poor) | -0.140 | 0.243 | | -0.58 | 0.56 | 0.022 | 0.363 | 0.06 | 0.95 |  |
| Children born | -0.031 | 0.028 | | -1.13 | 0.26 | -0.025 | 0.030 | -0.84 | 0.40 |  |
| Temperature | -0.011 | 0.057 | | -0.19 | 0.85 | 0.001 | 0.060 | 0.01 | 0.99 |  |
| Social class (rich) : children born | 0.000 |  | |  |  | 0.000 |  |  |  |  |
| Social class (poor) : children born | 0.035 | 0.028 | | 1.24 | 0.22 | -0.007 | 0.075 | -0.09 | 0.93 |  |
| Children born : temperature | 0.009 | 0.009 | | 0.94 | 0.35 | 0.006 | 0.010 | 0.64 | 0.52 |  |
| Social class (rich) : temperature | 0.000 |  | |  |  | 0.000 |  |  |  |  |
| Social class (poor) : temperature | 0.060 | 0.070 | | 0.86 | 0.39 | 0.002 | 0.120 | 0.01 | 0.99 |  |
| Social class (rich) : children born : temperature | - | - | | - | - | 0.000 |  |  |  |  |
| Social class (poor) : children born : temperature | - | - | | - | - | 0.016 | 0.026 | 0.60 | 0.55 |  |
| *Random effects* | Variance (SD) | |  |  |  | Variance (SD) |  |  |  |  |
| Maternal ID | 0.12 (0.35) |  | |  |  | 0.13 (0.35) |  |  |  |  |
| Birth year | 0.02 (0.14) |  | |  |  | 0.02 (0.13) |  |  |  |  |

There was linear association between number of children survived to adulthood and probability of survival (Hypothesis 1: hazard 0.94; 95% confidence interval 0.91 – 0.96; P<0.001). Additional child who survived to adulthood was associated with 6% lower probability of mother’s mortality. There was also a quadratic association between number of children survived and maternal survival (Hypothesis 2: linear hazard 0.86; 95% confidence interval 0.80 – 0.92; P<0.001, quadratic hazard 1.01; 95% confidence interval 1.00 – 1.02; P<0.01). Women had lower mortality risk up to seven children and then mortality risk started to increase.

**Table D.** A mixed-effects Cox regression model of the effects of number of children survived to adulthood and early-life environment – spring temperature - on maternal survival. Model 2 shows results for predictions of Hypothesis 3 and Model 2 a shows results for predictions of Hypothesis 4. β = Cox regression coefficient; s.e. (β) standard error of Cox regression coefficient

| **Variable** | **β** | **s.e. (β)** | | **z-value** | **p-value** | **β** | **s.e. (β)** | **z-value** | **p-value** |  |
| --- | --- | --- | --- | --- | --- | --- | --- | --- | --- | --- |
|  | *Model 2* | | | | | *Model 2a* | | | | |
| *Fixed effects* |  |  | |  |  |  |  |  |  |  |
| Birth status (singleton) | 0.000 |  | |  |  | 0.000 |  |  |  |  |
| Birth status (twin) | -0.081 | 0.133 | | -0.61 | 0.54 | -0.085 | 0.133 | -0.64 | 0.52 |  |
| Birth order (first) | 0.000 |  | |  |  | 0.000 |  |  |  |  |
| Birth order (subsequent) | -0.132 | 0.065 | | -2.03 | 0.04 | -0.135 | 0.065 | -2.08 | 0.04 |  |
| Parish, Hiittinen | 0.000 |  | |  |  | 0.000 |  |  |  |  |
| Parish, Ikaalinen | 0.116 | 0.087 | | 1.34 | 0.18 | 0.116 | 0.087 | 1.34 | 0.18 |  |
| Parish, Kustavi | 0.248 | 0.102 | | 2.44 | 0.02 | 0.253 | 0.102 | 2.48 | 0.01 |  |
| Parish, Rymättylä | 0.264 | 0.116 | | 2.27 | 0.02 | 0.258 | 0.116 | 2.22 | 0.03 |  |
| Parish, Tyrvää | 0.220 | 0.101 | | 2.18 | 0.03 | 0.217 | 0.101 | 2.16 | 0.03 |  |
| Social class (rich) | 0.000 |  | |  |  | 0.000 |  |  |  |  |
| Social class (poor) | -0.007 | 0.256 | | -0.03 | 0.98 | 0.175 | 0.415 | 0.42 | 0.67 |  |
| Children 15yrs | -0.131 | 0.105 | | -1.25 | 0.21 | -0.094 | 0.117 | -0.80 | 0.42 |  |
| Children 15yrs^2 | 0.002 | 0.013 | | 0.18 | 0.86 | -0.000 | 0.014 | -0.01 | 0.99 |  |
| Temperature | -0.019 | 0.066 | | -0.28 | 0.78 | 0.012 | 0.073 | 0.16 | 0.87 |  |
| Social class (rich) : children 15yrs | 0.000 |  | |  |  | 0.000 |  |  |  |  |
| Social class (poor) : children 15yrs | -0.107 | 0.096 | | -1.12 | 0.26 | -0.002 | 0.300 | -0.01 | 0.99 |  |
| Social class (rich) : children 15yrs^2 | 0.000 |  | |  |  | 0.000 |  |  |  |  |
| Social class (poor) : children 15yrs^2 | 0.019 | 0.014 | | 1.35 | 0.18 | -0.029 | 0.049 | -0.60 | 0.55 |  |
| Children 15yrs : temperature | -0.006 | 0.035 | | -0.17 | 0.87 | -0.018 | 0.040 | -0.46 | 0.64 |  |
| Children 15yrs^2 : temperature | 0.003 | 0.004 | | 0.72 | 0.47 | 0.004 | 0.005 | 0.82 | 0.41 |  |
| Social class (rich) : temperature | 0.000 |  | |  |  | 0.000 |  |  |  |  |
| Social class (poor) : temperature | 0.058 | 0.073 | | 0.81 | 0.42 | -0.004 | 0.136 | -0.03 | 0.98 |  |
| Social class (rich) : children 15yrs : temperature | - | - | | - | - | 0.000 |  |  |  |  |
| Social class (poor) : children 15yrs : temperature | - | - | | - | - | -0.047 | 0.105 | -0.45 | 0.65 |  |
| Social class (rich) : children 15yrs^2 : temperature | - | - | | - | - | 0.000 |  |  |  |  |
| Social class (poor) : children 15yrs^2 : temperature | - | - | | - | - | 0.021 | 0.018 | 1.13 | 0.26 |  |
| *Random effects* | Variance (SD) | |  |  |  | Variance (SD) |  |  |  |  |
| Maternal ID | 0.16 (0.40) |  | |  |  | 0.16 (0.40) |  |  |  |  |
| Birth year | 0.01 (0.11) |  | |  |  | 0.01 (0.11) |  |  |  |  |

**Infant mortality**

There was neither linear (Hypothesis 1: hazard 0.99, 95% confidence interval 0.97 – 1.01, P = 0.34) nor quadratic association between number of children born and survival probability at each time step (Hypothesis 2: hazard 1.00, 95% confidence interval 0.99 – 1.01, P = 0.70)

**Table E.** A mixed-effects Cox regression model of the effects of number of children born and early-life environment – infant mortality - on maternal survival. Model 1 shows results for predictions of Hypothesis 3 and Model 1a shows results for predictions of Hypothesis 4. β = Cox regression coefficient; s.e. (β) standard error of Cox regression coefficient

| **Variable** | **β** | **s.e. (β)** | | **z-value** | **p-value** | **β** | **s.e. (β)** | **z-value** | **p-value** |  |
| --- | --- | --- | --- | --- | --- | --- | --- | --- | --- | --- |
|  | *Model 1* | | | | | *Model 1a* | | | | |
| *Fixed effects* |  |  | |  |  |  |  |  |  |  |
| Birth status (singleton) | 0.000 |  | |  |  | 0.000 |  |  |  |  |
| Birth status (twin) | -0.154 | 0.144 | | -1.07 | 0.29 | -0.168 | 0.144 | -1.16 | 0.24 |  |
| Birth order (first) | 0.000 |  | |  |  | 0.000 |  |  |  |  |
| Birth order (subsequent) | -0.100 | 0.066 | | -1.51 | 0.13 | -0.098 | 0.066 | -1.47 | 0.14 |  |
| Parish, Hiittinen | 0.000 |  | |  |  | 0.000 |  |  |  |  |
| Parish, Ikaalinen | 0.036 | 0.095 | | 0.38 | 0.70 | 0.039 | 0.095 | 0.41 | 0.68 |  |
| Parish, Kustavi | 0.166 | 0.121 | | 1.37 | 0.17 | 0.167 | 0.121 | 1.38 | 0.17 |  |
| Parish, Rymättylä | 0.185 | 0.123 | | 1.50 | 0.13 | 0.186 | 0.123 | 1.51 | 0.13 |  |
| Parish, Tyrvää | 0.164 | 0.112 | | 1.46 | 0.14 | 0.169 | 0.112 | 1.51 | 0.13 |  |
| Social class (rich) | 0.000 |  | |  |  | 0.000 |  |  |  |  |
| Social class (poor) | 0.177 | 0.276 | | 0.64 | 0.52 | 0.692 | 0.447 | 1.55 | 0.12 |  |
| Children born | -0.016 | 0.031 | | -0.53 | 0.60 | -0.003 | 0.032 | -0.09 | 0.93 |  |
| Infant mortality | 0.287 | 1.019 | | 0.28 | 0.78 | 0.666 | 1.043 | 0.64 | 0.52 |  |
| Social class (rich) : children born | 0.000 |  | |  |  | 0.000 |  |  |  |  |
| Social class (poor) : children born | 0.032 | 0.028 | | 1.13 | 0.26 | -0.103 | 0.096 | -1.07 | 0.29 |  |
| Children born : infant mortality | 0.015 | 0.165 | | 0.09 | 0.93 | -0.062 | 0.174 | -0.36 | 0.72 |  |
| Social class (rich) : infant mortality | 0.000 |  | |  |  | 0.000 |  |  |  |  |
| Social class (poor) : infant mortality | -1.054 | 1.423 | | -0.74 | 0.46 | -4.140 | 2.558 | -1.62 | 0.11 |  |
| Social class (rich) : children born : infant mortality | - | - | | - | - |  |  |  |  |  |
| Social class (poor) : children born : infant mortality | - | - | | - | - | 0.814 | 0.552 | 1.47 | 0.14 |  |
| *Random effects* | Variance (SD) | |  |  |  | Variance (SD) |  |  |  |  |
| Maternal ID | 0.11 (0.33) |  | |  |  | 0.10 (0.32) |  |  |  |  |
| Birth year | 0.01 (0.12) |  | |  |  | 0.01 (0.12) |  |  |  |  |

There was linear association between number of children survived to adulthood and probability of survival (Hypothesis 1: hazard 0.93; 95% confidence interval 0.91 – 0.96; P<0.001). Additional child who survived to adulthood was associated with 6% lower probability of mother’s mortality. There was also a quadratic association between number of children survived and maternal survival (Hypothesis 2: linear hazard 0.86; 95% confidence interval 0.80 – 0.92; P<0.001, quadratic hazard 1.01; 95% confidence interval 1.00 – 1.02; P=0.01). Women had lower mortality risk up to seven children and then mortality risk started to increase.

**Table F.** A mixed-effects Cox regression model of the effects of number of children survived to adulthood and early-life environment – infant mortality - on maternal survival. Model 2 shows results for predictions of Hypothesis 3 and Model 2 a shows results for predictions of Hypothesis 4. β = Cox regression coefficient; s.e. (β) standard error of Cox regression coefficient

| **Variable** | **β** | **s.e. (β)** | | **z-value** | **p-value** | **β** | **s.e. (β)** | **z-value** | **p-value** |  |
| --- | --- | --- | --- | --- | --- | --- | --- | --- | --- | --- |
|  | *Model 2* | | | | | *Model 2a* | | | | |
| *Fixed effects* |  |  | |  |  |  |  |  |  |  |
| Birth status (singleton) | 0.000 |  | |  |  | 0.000 |  |  |  |  |
| Birth status (twin) | -0.133 | 0.146 | | -0.91 | 0.36 | -0.125 | 0.147 | -0.85 | 0.39 |  |
| Birth order (first) | 0.000 |  | |  |  | 0.000 |  |  |  |  |
| Birth order (subsequent) | -0.130 | 0.067 | | -1.92 | 0.05 | -0.128 | 0.068 | -1.89 | 0.06 |  |
| Parish, Hiittinen | 0.000 |  | |  |  | 0.000 |  |  |  |  |
| Parish, Ikaalinen | 0.057 | 0.097 | | 0.59 | 0.56 | 0.053 | 0.097 | 0.55 | 0.58 |  |
| Parish, Kustavi | 0.144 | 0.124 | | 1.16 | 0.25 | 0.140 | 0.124 | 1.12 | 0.26 |  |
| Parish, Rymättylä | 0.208 | 0.126 | | 1.65 | 0.10 | 0.203 | 0.126 | 1.61 | 0.11 |  |
| Parish, Tyrvää | 0.161 | 0.114 | | 1.41 | 0.16 | 0.163 | 0.115 | 1.42 | 0.16 |  |
| Social class (rich) | 0.000 |  | |  |  | 0.000 |  |  |  |  |
| Social class (poor) | 0.363 | 0.289 | | 1.25 | 0.21 | 0.420 | 0.474 | 0.88 | 0.38 |  |
| Children 15yrs | -0.052 | 0.121 | | -0.43 | 0.67 | -0.048 | 0.130 | -0.37 | 0.71 |  |
| Children 15yrs^2 | -0.008 | 0.016 | | -0.49 | 0.63 | -0.006 | 0.017 | -0.36 | 0.72 |  |
| Infant mortality | 0.531 | 1.221 | | 0.44 | 0.66 | 0.740 | 1.308 | 0.57 | 0.57 |  |
| Social class (rich) : children 15yrs | 0.000 |  | |  |  | 0.000 |  |  |  |  |
| Social class (poor) : children 15yrs | -0.130 | 0.097 | | -1.33 | 0.18 | 0.102 | 0.364 | 0.28 | 0.78 |  |
| Social class (rich) : children 15yrs^2 | 0.000 | 0.014 | | 1.48 | 0.14 | 0.000 |  |  |  |  |
| Social class (poor) : children 15yrs^2 | 0.021 |  | |  |  | -0.045 | 0.060 | -0.75 | 0.45 |  |
| Children 15yrs : infant mortality | -0.488 | 0.649 | | -0.75 | 0.45 | -0.509 | 0.706 | -0.72 | 0.47 |  |
| Children 15yrs^2 : infant mortality | 0.096 | 0.087 | | 1.10 | 0.27 | 0.086 | 0.092 | 0.93 | 0.35 |  |
| Social class (rich) : infant mortality | 0.000 |  | |  |  | 0.000 |  |  |  |  |
| Social class (poor) : infant mortality | -1.211 | 1.470 | | -0.82 | 0.41 | -1.508 | 2.642 | -0.57 | 0.57 |  |
| Social class (rich) : children 15yrs : infant mortality | - | - | | - | - | 0.000 |  |  |  |  |
| Social class (poor) : children 15yrs : infant mortality | - | - | | - | - | -1.451 | 2.088 | -0.70 | 0.49 |  |
| Social class (rich) : children 15yrs^2 : infant mortality | - | - | | - | - | 0.000 |  |  |  |  |
| Social class (poor) : children 15yrs^2: infant mortality | - | - | | - | - | 0.413 | 0.353 | 1.17 | 0.24 |  |
| *Random effects* | Variance (SD) | |  |  |  | Variance (SD) |  |  |  |  |
| Maternal ID | 0.14 (0.37) |  | |  |  | 0.14 (0.37) |  |  |  |  |
| Birth year | 0.01 (0.09) |  | |  |  | 0.01 (0.09) |  |  |  |  |
